# Bidirectional longitudinal associations between low anterior resection syndrome and stigma in patients with postoperative LARS: a cross-lagged panel study

**DOI:** 10.3389/fpubh.2026.1856613

**Published:** 2026-07-29

**Authors:** Xiangdong Hu, Qiushi Yin, Mei Cao, Changcui Liang, Hui Yang

**Affiliations:** 1 Department of General Surgery, Lu'an Hospital of Anhui Medical University (Lu'an People's Hospital), Lu'an, China; 2 Graduate College, Wannan Medical University, Wuhu, China; 3 Department of Urology, Affiliated Hospital of West Anhui Health Vocational College, Lu'an, China; 4 Residency Training and Education Department, Lu'an Hospital of Anhui Medical University (Lu'an People's Hospital), Lu'an, China

**Keywords:** cross-lagged panel model, longitudinal study, low anterior resection syndrome (LARS), mental health, rectal cancer, stigma

## Abstract

**Objective:**

To investigate the bidirectional longitudinal associations between low anterior resection syndrome (LARS) and stigma among patients with postoperative LARS following sphincter-preserving surgery for rectal cancer.

**Methods:**

A total of 201 patients with LARS after sphincter-preserving surgery for rectal cancer were recruited using convenience sampling between March 2024 and June 2025. Follow-up was completed in December 2025. Data were collected at 1 month (T1), 3 months (T2), and 6 months (T3) after discharge using the Low Anterior Resection Syndrome Score (LARS) and the Social Impact Scale (SIS). Of the enrolled participants, 181 with complete data across all follow-up assessments were included in the final cross-lagged panel model analysis.

**Results:**

LARS scores decreased from 37.28 ± 1.61 at T1 to 28.64 ± 2.86 at T2 and 27.52 ± 2.38 at T3. Stigma scores declined from 67.47 ± 4.06 at T1 to 60.52 ± 3.96 at T2 and 53.40 ± 4.22 at T3 (*P* < 0.05). Cross-lagged analysis revealed significant reciprocal longitudinal associations between LARS and stigma. Higher LARS scores were associated with higher subsequent stigma scores, while higher stigma scores were associated with higher subsequent LARS scores (all *P* < 0.05). Bidirectional longitudinal associations were observed between LARS and stigma.

**Conclusion:**

LARS and stigma were longitudinally associated among patients with postoperative LARS following sphincter-preserving surgery for rectal cancer. These findings suggest that both symptom burden and psychosocial adaptation should be considered during postoperative follow-up.

## Introduction

Rectal cancer is one of the most common malignancies of the digestive system worldwide, with high incidence and mortality rates, and has become a major public health concern (1, 2). In recent years, epidemiological studies have further demonstrated that the global burden of colorectal cancer continues to increase, particularly in developing countries (3, 4). With the improvement of screening strategies and the optimization of multidisciplinary treatment approaches, survival outcomes of rectal cancer patients have significantly improved (5). Meanwhile, increasing attention has been paid to postoperative quality of life, and functional preservation has gradually become an important consideration in treatment decision-making (6).

In this context, sphincter-preserving surgery, particularly low anterior resection, has become a major treatment option for patients with mid- and low-rectal cancer. This approach allows patients to avoid permanent stoma while preserving anal function to a certain extent (7). However, intraoperative nerve injury and alterations in rectal reservoir function often result in the development of low anterior resection syndrome (low anterior resection syndrome, LARS) after surgery (8, 9). In recent years, international consensus has systematically defined LARS and its clinical manifestations, establishing it as an important indicator for evaluating postoperative functional recovery (10, 11).

Studies have shown that approximately 70%−90% of patients experience varying degrees of LARS after surgery (12). The syndrome is mainly characterized by increased bowel frequency, urgency, fecal incontinence, and incomplete evacuation, which severely affect patients' daily life (13, 14). Further studies indicate that LARS is not only highly prevalent but also persistent, with some patients experiencing symptoms for several years (15). Long-term follow-up studies have demonstrated that LARS significantly reduces quality of life and exerts sustained negative effects on social functioning and psychological wellbeing (16).

Importantly, the impact of LARS extends beyond physical symptoms and profoundly affects patients' psychological and social adaptation. Frequent bowel dysfunction and unpredictable bowel patterns often lead to embarrassment, uncertainty in social situations, and subsequent avoidance behaviors, contributing to feelings of shame and low self-esteem (17). Over time, some patients internalize these experiences, forming negative self-perceptions and perceiving themselves as “abnormal.” During this process, patients may also perceive potential prejudice and discrimination from others, which constitutes a complex psychological experience known as stigma (18).

Stigma is a multidimensional psychosocial response encompassing internalized shame, perceived social rejection, and social isolation, and has been shown to be closely associated with mental health outcomes in patients with chronic diseases and cancer (19). Previous studies have indicated that higher levels of stigma not only exacerbate anxiety and depression but also impair social functioning and role recovery. Furthermore, stigma may negatively affect the rehabilitation process by reducing treatment adherence and limiting the effective utilization of social support (19). In patients with rectal cancer, it has been reported that the severity of LARS symptoms is positively correlated with the level of stigma (6). Alleviation of LARS symptoms may contribute to a reduction in stigma and improvement in quality of life (20). However, most of these studies are based on cross-sectional designs, which primarily reveal associations between variables but fail to explain their dynamic interactions over time.

From a theoretical perspective, the relationship between LARS and stigma may be understood through the symptom–cognition–emotion pathway, which suggests that physical symptoms may influence cognitive appraisal and emotional responses, as well as the stress and coping framework. Persistent bowel dysfunction, including fecal urgency, incontinence, and frequent bowel movements, may disrupt daily activities and social participation, leading patients to perceive themselves as different from others and increasing feelings of shame, embarrassment, and social devaluation. These cognitive and emotional responses may contribute to the development of stigma (21).

Conversely, stigma may influence subsequent symptom experiences through behavioral and psychological mechanisms. Patients experiencing higher levels of stigma may be less likely to seek professional support, communicate openly about symptoms, or actively engage in self-management and rehabilitation. Stigma-related psychological distress may also promote social withdrawal and impair long-term adaptation to cancer survivorship (22). Therefore, a dynamic reciprocal relationship between LARS and stigma may exist over time. However, empirical evidence regarding the temporal direction and reciprocal nature of this relationship remains limited.

The cross-lagged panel model is a classical longitudinal analytical method that allows for the examination of temporal relationships and directional associations between variables while controlling for their stability over time (23). This method has been widely applied in psychology and medical research to investigate dynamic interactions between variables. However, there is still a lack of studies using this approach to systematically explore the relationship between LARS and stigma in patients after sphincter-preserving surgery for rectal cancer.

Therefore, this study aimed to investigate the temporal trends and bidirectional relationship between low anterior resection syndrome (LARS) and stigma in patients after sphincter-preserving surgery for rectal cancer using a longitudinal design. A cross-lagged panel model was constructed to clarify the directionality of these associations over time. Importantly, from a nursing perspective, this study seeks to provide evidence for early identification of high-risk patients and to inform the development of targeted, nurse-led interventions that simultaneously address symptom management and psychosocial adaptation, ultimately improving postoperative recovery and quality of life.

## Methods

### Participants

According to sample size estimation principles for structural equation modeling, the required sample size should exceed 200 cases, with a ratio of at least 10–15 participants per observed variable. In this study, a maximum of 11 variables were included. Considering a potential attrition rate of 15%, the minimum required sample size was estimated to range from 127 to 190 participants.

A total of 201 patients with LARS after sphincter-preserving surgery for rectal cancer were recruited between March 2024 and June 2025. Follow-up was completed in December 2025. Data were collected at 1 month (T1), 3 months (T2), and 6 months (T3) after discharge using the Low Anterior Resection Syndrome Score (LARS) and the Social Impact Scale (SIS). A cross-lagged panel model was constructed using AMOS 24.0 to examine the temporal and reciprocal relationships between LARS and stigma.

The inclusion criteria were as follows: (1) age ≥18 years; (2) pathologically confirmed rectal cancer treated with sphincter-preserving surgery and diagnosed with LARS; and (3) ability to understand the study and willingness to participate, with written informed consent provided. The exclusion criteria included: (1) a history of other malignant colorectal tumors or recent diagnosis of other malignancies; (2) presence of intestinal obstruction, perforation, or bleeding requiring emergency surgery; and (3) a history of severe psychiatric disorders. Participants were excluded if they had incomplete baseline data or withdrew from the study or failed to complete follow-up for any reason.

This study was approved by the institutional ethics committee (Approval No. 2024LLKS-KY-028; approved on February 26, 2024), and all participants provided written informed consent prior to enrollment.

### Measures

#### General information questionnaire

A structured questionnaire designed by the research team was used to collect demographic and clinical data, including gender, age, marital status, education level, income, body mass index (BMI), history of chronic disease, tumor metastasis, and tumor stage.

#### Low anterior resection syndrome score (LARS)

The LARS score, developed by Emmertsen et al. (24), was used to assess bowel dysfunction in patients after rectal cancer surgery. The scale evaluates five core symptoms: incontinence for flatus, incontinence for liquid stools, frequency of bowel movements, urgency, and clustering. The Chinese version of the LARS score was translated and validated by Cao et al. (25), demonstrating good reliability and validity. Yan et al. (26) further applied the scale to assess bowel dysfunction severity in Chinese patients. The total score ranges from 0 to 42, with higher scores indicating more severe dysfunction. Scores ≥30 indicate major LARS, 21–29 indicate minor LARS, and ≤ 20 indicate no LARS. The scale has a sensitivity of 93.8% and a specificity of 76.7%. The LARS score is a symptom-based clinical index designed to assess the severity of bowel dysfunction rather than a reflective psychometric scale. Therefore, internal consistency reliability was not evaluated in the present study.

#### Social impact scale (SIS)

Stigma was assessed using the Social Impact Scale (SIS), originally developed by Fife et al. (27) and later adapted for Chinese populations by Shen et al. (28). The revised version includes four dimensions: social rejection, financial insecurity, internalized shame, and social isolation, comprising a total of 20 items.

The total score ranges from 20 to 80, with higher scores indicating greater stigma. Scores of 20–39 indicate low stigma, 40–59 indicate moderate stigma, and 60–80 indicate high stigma. The scale demonstrated good reliability and validity, with a Cronbach's α coefficient of 0.943 and a KMO value of 0.785 (*P* < 0.05).

The SIS has demonstrated satisfactory reliability and validity in previous validation studies. As the LARS score is a symptom-based clinical index rather than a reflective psychometric scale, internal consistency reliability was not assessed.

### Data collection

Data were collected at three time points: 1 month (T1), 3 months (T2), and 6 months (T3) after discharge. At T1, trained researchers explained the study procedures using standardized instructions. After obtaining consent, paper questionnaires were distributed. Participants completed the questionnaires independently, with assistance provided when necessary. Completed questionnaires were collected on site.

At T2 and T3, data were collected through follow-up visits, telephone interviews, or outpatient reviews. All returned questionnaires were checked for completeness and logical consistency. Data were double-entered into a computer system by two independent researchers.

A total of 201 eligible participants were enrolled in the study. Six participants were excluded because of incomplete questionnaires at T1, resulting in 195 participants completing the first assessment. During follow-up, eight participants were lost between T1 and T2, and six participants were lost between T2 and T3. Consequently, 187 participants completed the T2 assessment and 181 participants completed the T3 assessment. Only participants with complete data across all three follow-up assessments were included in the final cross-lagged panel model analysis (*n* = 181).

### Statistical analysis

Data were analyzed using SPSS version 27.0. Normally distributed continuous variables are presented as mean ± SD. Group comparisons were performed using independent-samples *t*-tests or one-way analysis of variance. Changes in LARS and stigma scores across T1, T2, and T3 were analyzed using repeated-measures analysis of variance (ANOVA), and pairwise comparisons were conducted when appropriate. Pearson correlation analysis was used to examine associations between variables.

A cross-lagged panel model was constructed using AMOS version 24.0 to examine the temporal and reciprocal relationships between LARS and stigma. The model included autoregressive paths and cross-lagged paths across adjacent assessment points. Correlations between LARS and SIS measured at the same assessment point were estimated. A residual covariance between the error terms of LARS at T2 and T3 was estimated based on modification indices and retained because it improved model fit and was theoretically plausible for repeated measurements of the same construct. No equality constraints were imposed on the cross-lagged paths. Model fit was evaluated using chi-square (χ^2^), degrees of freedom (df), chi-square/degrees of freedom (CMIN/DF), adjusted goodness-of-fit index (AGFI), incremental fit index (IFI), Tucker–Lewis index (TLI), comparative fit index (CFI), and root mean square error of approximation (RMSEA) with its 90% confidence interval (CI). A model was considered to have an acceptable fit when CMIN/DF < 3, AGFI, IFI, TLI, and CFI > 0.90, and RMSEA < 0.08. Statistical significance was set at *P* < 0.05.

## Results

## Participant flow

**Figure 1:**
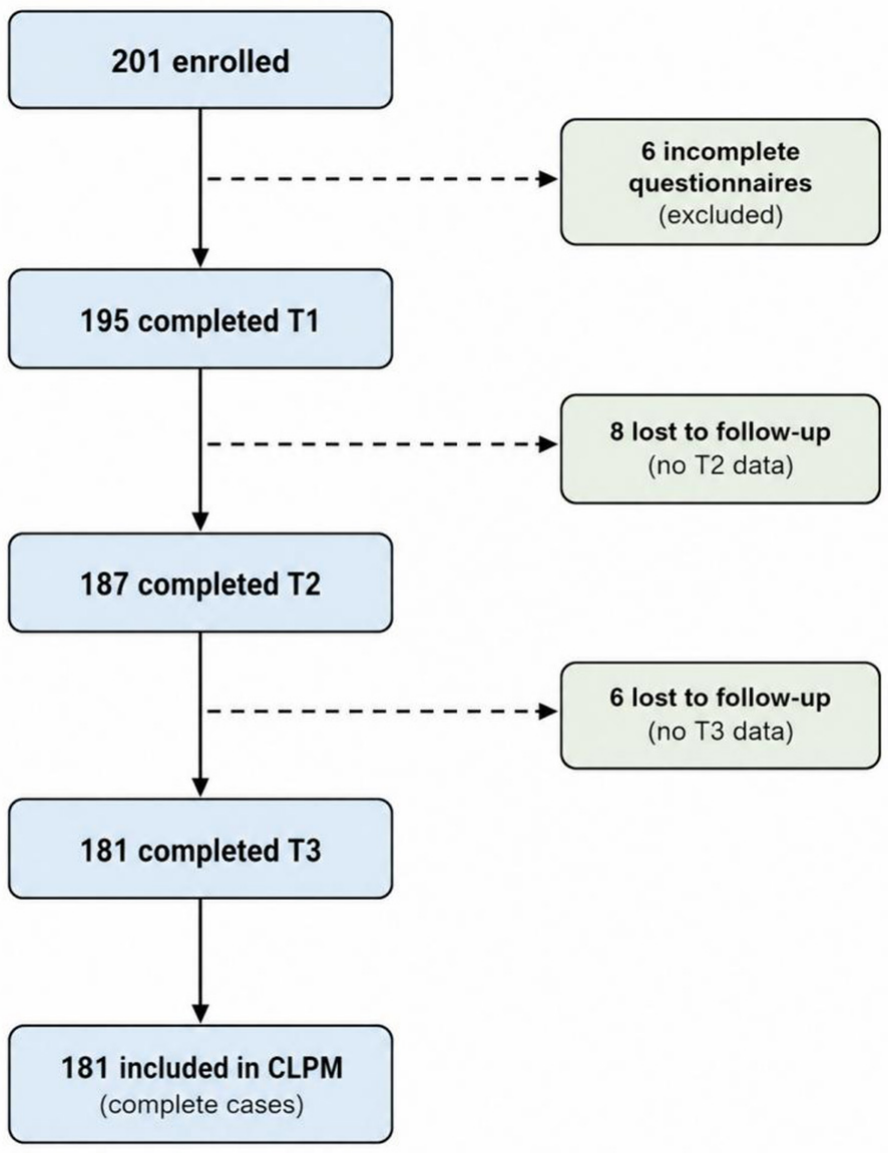
Flow diagram of participant recruitment, follow-up, and retention. CLPM, cross-lagged panel model; T1, 1 month after discharge; T2, 3 months after discharge; T3, 6 months after discharge.

A total of 201 patients were enrolled in the study. Of these, 195 completed the T1 assessment, 187 completed the T2 assessment, and 181 completed the T3 assessment. Ultimately, 181 participants with complete longitudinal data were included in the cross-lagged panel model analysis (Figure 1). The participant flow is presented in Figure 1.

## Attrition analysis

Of the 201 participants initially enrolled, 181 completed all follow-up assessments and were included in the final analysis, whereas 20 participants were lost to follow-up. Attrition analysis was conducted to compare participants who completed all assessments with those who were lost during follow-up. No statistically significant differences were observed in baseline demographic or clinical characteristics, including age, sex, education level, BMI, tumor stage, baseline LARS score, and baseline SIS score (all *P* > 0.05). These findings suggest that attrition was unlikely to introduce substantial bias into the study results.

## General characteristics and univariate analysis

The results of the univariate analysis showed that there were no statistically significant differences in LARS scores or stigma scores at T1 across different groups (*P* > 0.05), as presented in Table 1.

**Table 1:** Univariate analysis of LARS and stigma at T1.

Variable	*n* (%)	LARS score at T1 (mean ±SD)	t/F	*P* value	Stigma score at T1 (mean ±SD)	t/F	*P* value
Gender			0.307	0.580		1.541	0.216
Male	122 (67.4)	37.33 ± 1.68			67.73 ± 3.85		
Female	59 (32.6)	37.19 ± 1.46			66.93 ± 4.43		
Age			1.190	0.277		2.224	0.138
< 60 years	60 (33.1)	37.47 ± 1.59			66.83 ± 4.39		
≥60 years	121 (66.9)	37.19 ± 1.61			67.79 ± 3.86		
Marital status			2.420	0.122		1.137	0.288
Unmarried	16 (8.8)	36.69 ± 1.92			66.44 ± 4.35		
Married	165 (91.2)	37.34 ± 1.57			67.57 ± 4.03		
Education			2.714	0.101		2.255	0.135
Junior or below	127 (70.2)	37.41 ± 1.55			67.76 ± 3.81		
Senior or above	54 (29.8)	36.98 ± 1.72			66.78 ± 4.54		
Income (RMB)			0.489	0.614		2.605	0.077
< 3000	41 (22.7)	37.29 ± 1.63			68.71 ± 4.61		
3000–5000	103 (56.9)	37.36 ± 1.61			67.02 ± 3.87		
>5000	37 (20.4)	37.05 ± 1.60			67.35 ± 3.73		
BMI			0.139	0.710		2.687	0.103
< 24	130 (71.8)	37.25 ± 1.56			67.16 ± 3.96		
≥24	51 (28.2)	37.35 ± 1.74			68.25 ± 4.23		
Chronic disease			1.980	0.161		2.479	0.117
Yes	103 (56.9)	37.14 ± 1.70			67.06 ± 3.77		
No	78 (43.1)	37.47 ± 1.46			68.01 ± 4.37		
Metastasis			1.432	0.233		0.804	0.371
Yes	31 (17.1)	36.97 ± 1.87			68.06 ± 4.21		
No	150 (82.9)	37.35 ± 1.55			67.35 ± 4.03		
Tumor stage			1.543	0.205		0.401	0.752
T1	25 (13.8)	37.56 ± 1.53			68.08 ± 5.02		
T2	59 (32.6)	37.54 ± 1.51			67.68 ± 3.86		
T3	81 (44.8)	37.01 ± 1.68			67.21 ± 3.91		
T4	16 (8.8)	37.28 ± 1.61			67.06 ± 4.07		

Values are presented as mean ± SD or n (%).

LARS, Low Anterior Resection Syndrome; BMI, body mass index.

Repeated-measures ANOVA demonstrated significant time effects for both LARS and stigma. Specifically, a significant time effect was observed for LARS (F (2, 360) = 632.368, *P* < 0.001) and stigma (F (2, 360) = 358.885, *P* < 0.001). Pairwise comparisons indicated that LARS scores differed significantly between T1 and T2, T1 and T3, and T2 and T3 (all *P* < 0.05). Similarly, stigma scores differed significantly between T1 and T2, T1 and T3, and T2 and T3 (all *P* < 0.05).

Pearson correlation analysis indicated that LARS was positively correlated with stigma at T1, T2, and T3 (all *P* < 0.05) (Table 2).

**Table 2:** Correlations between LARS and stigma across time points.

Variable	Mean ±SD	T1 LARS	T2 LARS	T3 LARS	T1 SIS	T2 SIS	T3 SIS
T1 LARS	37.28 ± 1.61	1.000					
T2 LARS	28.64 ± 2.86^a^	0.499^**^	1.000				
T3 LARS	27.52 ± 2.38^a^^b^	0.514^**^	0.407^**^	1.000			
T1 SIS	67.47 ± 4.06	0.566^**^	0.587^**^	0.543^**^	1.000		
T2 SIS	60.52 ± 3.96^a^	0.474^**^	0.458^**^	0.479^**^	0.579^**^	1.000	
T3 SIS	53.40 ± 4.22^a^^b^	0.178^*^	0.392^**^	0.228^**^	0.333^**^	0.516^**^	1.000

Values are presented as mean ± SD or Pearson correlation coefficients.

LARS, Low Anterior Resection Syndrome; SIS, Social Impact Scale.

^a^*P* < 0.05 compared with T1.

^b^*P* < 0.05 compared with T2. ^*^P < 0.05. ^**^P < 0.01.

## Cross-lagged analysis of LARS and stigma

The cross-lagged panel model demonstrated a good model fit, with CMIN/DF < 3, AGFI, CFI, IFI, and TLI all >0.90, and RMSEA < 0.08 (Table 3).

**Table 3:** Model fit indices of the cross-lagged panel model.

Model	χ^2^	df	*P*	CMIN/DF	AGFI	CFI	IFI	TLI	RMSEA (90% CI)
LARS–SIS	9.797	5	0.081	1.959	0.924	0.987	0.988	0.962	0.073 (0.000–0.141)

χ^2^= chi-square statistic; df, degrees of freedom; CMIN/DF, chi-square/degrees of freedom; AGFI, adjusted goodness-of-fit index; IFI, incremental fit index; TLI, Tucker–Lewis index; CFI, comparative fit index; RMSEA, root mean square error of approximation; CI, confidence interval.

**Figure 2:**
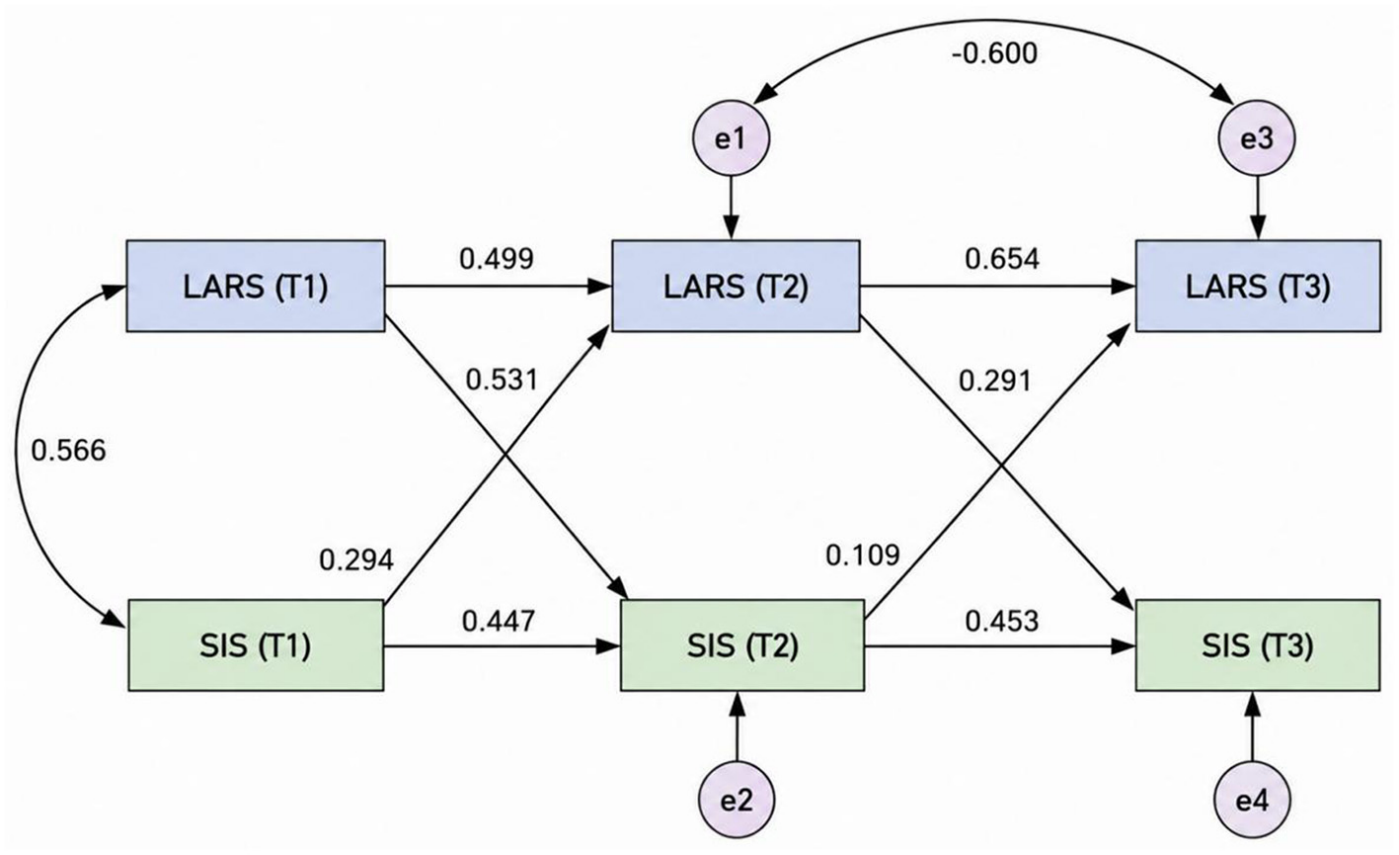
Cross-lagged panel model of the reciprocal relationships between low anterior resection syndrome (LARS) and stigma across three assessment points. Legend: LARS, Low Anterior Resection Syndrome; SIS, Social Impact Scale. T1, T2, and T3 represent 1 month, 3 months, and 6 months after discharge, respectively. Solid arrows represent autoregressive and cross-lagged effects. Curved double-headed arrows represent correlations. Values shown are standardized path coefficients (β). All displayed paths were statistically significant (*P* < 0.05). A residual covariance between the error terms of LARS at T2 and T3 was included to improve model fit.

Autoregressive effects were observed for both LARS and stigma. Specifically, LARS at T1 significantly predicted LARS at T2 (β = 0.499, *P* < 0.05), and LARS at T2 predicted LARS at T3 (β = 0.654, *P* < 0.05). Similarly, stigma at T1 predicted stigma at T2 (β = 0.447, *P* < 0.05), and stigma at T2 predicted stigma at T3 (β = 0.453, *P* < 0.05). Cross-lagged effects indicated a bidirectional relationship between LARS and stigma. LARS at T1 significantly predicted stigma at T2 (β = 0.531, *P* < 0.05), while stigma at T1 predicted LARS at T2 (β = 0.294, *P* < 0.05). Likewise, LARS at T2 predicted stigma at T3 (β = 0.291, *P* < 0.05), and stigma at T2 predicted LARS at T3 (β = 0.109, *P* < 0.05). All path coefficients were statistically significant (*P* < 0.05). The model is illustrated in Figure 2, and the detailed path coefficients are presented in Table 4.

**Table 4:** Unstandardized path coefficients from the cross-lagged panel model.

Path	Estimate	SE	CR	*P*
T2 LARS ← T1 LARS	0.499	0.105	4.755	< 0.001
T2 SIS ← T1 SIS	0.447	0.070	6.360	< 0.001
T2 SIS ← T1 LARS	0.531	0.177	2.995	0.003
T2 LARS ← T1 SIS	0.294	0.043	6.778	< 0.001
T3 LARS ← T2 LARS	0.654	0.115	5.685	< 0.001
T3 SIS ← T2 SIS	0.453	0.072	6.309	< 0.001
T3 LARS ← T2 SIS	0.109	0.044	2.465	0.014
T3 SIS ← T2 LARS	0.291	0.099	2.930	0.003

Estimates represent unstandardized path coefficients.

SE, standard error; CR, critical ratio.

## Cross-lagged panel model

The cross-lagged panel model demonstrated an acceptable fit to the data (χ^2^ = 9.797, df = 5, *P* = 0.081, CMIN/DF = 1.959, AGFI = 0.924, CFI = 0.987, IFI = 0.988, TLI = 0.962, RMSEA = 0.073, 90% CI: 0.000–0.141) (Table 3).

## Discussion

### Trends in LARS following sphincter-preserving surgery

The present study demonstrated that patients experienced severe LARS at 1 month after discharge, indicating substantial impairment of bowel function during the early postoperative stage. This finding is consistent with previous studies reporting high symptom burden shortly after sphincter-preserving surgery (29). The severity of early symptoms may be attributed to intraoperative nerve injury, reduced rectal reservoir capacity, and delayed recovery of anorectal function (30). Over time, LARS scores decreased significantly at 3 and 6 months, suggesting gradual functional recovery. This improvement may be explained by neural adaptation, recovery of sphincter control, and patients' increasing ability to self-manage bowel function (31, 32). However, despite this improvement, a considerable level of dysfunction persisted, indicating that LARS remains a long-term survivorship issue rather than a transient postoperative complication (15). From a clinical perspective, these findings highlight the importance of early symptom monitoring and proactive management strategies during the initial postoperative period. Timely interventions targeting bowel function may not only improve physical recovery but also reduce the downstream psychosocial burden associated with persistent symptoms.

### Trends in stigma among patients

At T1, patients exhibited high levels of stigma (67.47 ± 4.06), which is consistent with previous findings (33). Early postoperative stigma may arise from bowel dysfunction symptoms such as urgency, incontinence, and odor, which can lead to embarrassment and perceived social judgment (34). Furthermore, these symptoms may negatively affect patients' self-perception, contributing to internalized stigma (35).

Stigma levels decreased at T2 and T3, suggesting gradual psychological adaptation. This may be partly attributable to improvements in LARS symptoms, which reduce the frequency of socially distressing events. Additionally, patients may gradually develop self-management strategies, such as dietary adjustments and bowel regulation, thereby enhancing self-efficacy (36, 37). However, stigma remained elevated at 6 months, indicating that symptom improvement alone is insufficient to fully address psychological adaptation.

These findings underscore the importance of recognizing stigma as a significant component of the disease burden in rectal cancer survivorship. Addressing stigma requires not only symptom control but also targeted psychosocial support to facilitate long-term adaptation and reintegration into social roles.

### Bidirectional relationship between LARS and stigma

This study demonstrated a bidirectional predictive relationship between LARS and stigma over time. LARS at T1 predicted stigma at T2 (β = 0.531), while stigma at T1 predicted LARS at T2 (β = 0.294). Similar relationships were observed between T2 and T3 (β = 0.291 and β =0.109, respectively; all *P* < 0.05). These findings indicate that LARS and stigma interact dynamically over time.

Notably, bidirectional longitudinal associations were observed between LARS and stigma. Severe bowel dysfunction may be associated with higher levels of stigma by restricting social participation, impairing body image, and disrupting social roles. Previous studies have also reported an association between bowel dysfunction and stigma in patients with colorectal cancer (38).

Conversely, the association between stigma and subsequent LARS may be indirect. Higher levels of stigma may be associated with lower help-seeking behavior, poorer treatment adherence, and greater psychological stress, which may in turn be related to less favorable functional adaptation during recovery (39). These findings suggest that physical symptoms and psychosocial distress may be longitudinally associated and may co-occur over time.

From a clinical perspective, early symptom management is crucial, as it may not only improve physical outcomes but also prevent or mitigate subsequent psychological distress. At the same time, the reverse influence of stigma should not be overlooked, as persistent stigma may be associated with poorer adaptation during recovery.

Taken together, these findings suggest a dynamic reciprocal association between LARS and stigma. Bidirectional longitudinal associations were observed between LARS and stigma over time. However, these findings should be interpreted as temporal associations rather than evidence of causality.

These findings support a dynamic “symptom–psychosocial distress” interaction framework, in which physical dysfunction and stigma reinforce each other over time, forming a self-perpetuating cycle. This mechanism provides a more comprehensive understanding of the recovery process in rectal cancer survivors.

### Implications for clinical practice and public health

The findings of this study suggest that both symptom burden and psychosocial adaptation should be considered during postoperative follow-up for patients with postoperative LARS. Routine assessment of bowel dysfunction and stigma may help healthcare professionals identify patients who experience persistent physical and psychosocial difficulties (40).

Given the observed longitudinal associations between LARS and stigma, symptom management and psychosocial support may be considered as complementary components of postoperative care (41, 42). However, further research is needed to determine whether interventions targeting these factors can improve long-term outcomes.

From a public health perspective, the findings highlight the importance of addressing both functional and psychosocial challenges during cancer survivorship (43). Future studies should evaluate strategies to support long-term adaptation and quality of life among patients with postoperative LARS (44, 45).

## Conclusion

In conclusion, LARS and stigma were longitudinally and reciprocally associated among patients with postoperative LARS following sphincter-preserving surgery for rectal cancer. These findings suggest that both symptom burden and psychosocial adaptation should be considered during postoperative follow-up. Further studies are needed to determine whether interventions targeting these factors can improve long-term outcomes.

### Limitations and future directions

This study has several limitations. First, participants were recruited from a single center using convenience sampling, and only patients with postoperative LARS were included, which may limit the generalizability of the findings. Second, the follow-up period was relatively short, and all outcomes were assessed using self-reported questionnaires, which may be subject to reporting and measurement bias. Although no significant baseline differences were observed between retained and lost participants, the potential influence of attrition cannot be completely excluded.

Third, the cross-lagged panel model did not account for several potentially important clinical, treatment-related, and psychosocial factors, such as neoadjuvant chemoradiotherapy, temporary stoma status, surgical technique, postoperative complications, adjuvant therapy, social support, and self-management behaviors, which may have influenced the observed associations.

Finally, the traditional cross-lagged panel model cannot fully distinguish stable between-person differences from within-person changes over time, and longitudinal measurement invariance was not formally examined. Future studies should employ multicenter designs, longer follow-up periods, and more advanced longitudinal modeling approaches to further validate and extend the present findings.

## Data Availability

The raw data supporting the conclusions of this article will be made available by the authors, without undue reservation.
